# Decreased testosterone levels after caponization leads to abdominal fat deposition in chickens

**DOI:** 10.1186/s12864-018-4737-3

**Published:** 2018-05-09

**Authors:** Xiaoyan Cui, Huanxian Cui, Lu Liu, Guiping Zhao, Ranran Liu, Qinghe Li, Maiqing Zheng, Jie Wen

**Affiliations:** 1 Institute of Animal Sciences, Chinese Academy of Agricultural Science, Beijing, 100193 China; 2 State Key Laboratory of Animal Nutrition, Beijing, 100193 China; 3 Institute of Animal Science, Guangdong Academy of Agricultural Sciences, Guangzhou, 510640 China

**Keywords:** Capon, Testosterone, Lipid metabolism, Differential expression gene, PPAR pathway

## Abstract

**Background:**

Caponization results in reduced androgen levels, which leads to abdominal fat accumulation in capons. In this study, we sought to understand the molecular mechanisms behind this fat accumulation.

**Results:**

Abdominal fat (AF) content increased significantly (*P* < 0.05) and serum and AF testosterone levels decreased significantly (*P* < 0.05 or *P* < 0.01) after caponization. In AF tissue, 90 differentially expressed genes related to lipid metabolism were screened by gene expression profiling in caponized and sham-treated chickens. Among these, six representative genes were significantly up-regulated (*APOA1*, *SCD*, *FABP7*, *RXRG,* and *FADS2*) or down-regulated (*FABP3*) (*P* < 0.05 or *P* < 0.01) and were strongly associated with the PPAR pathway. In addition, cell junction pathways were also enriched. In vitro, Fat content was significantly lower in cells treated with testosterone compared with control cells (*P* < 0.01), and mRNA levels of *RXRG*, *FABP7,* and *FABP3* changed accordingly, confirming the effect of testosterone on fat deposition.

**Conclusions:**

The results of this study indicate that testosterone reduction likely regulates gene expression through PPAR and cell junction pathways resulting in increased fat accumulation. These results provide increase our understanding of the biological mechanisms by which caponization induces greater fat accumulation.

**Electronic supplementary material:**

The online version of this article (10.1186/s12864-018-4737-3) contains supplementary material, which is available to authorized users.

## Background

Capons are castrated male chickens using a technique called caponization. Caponized chicken meat is more tender, juicy, and flavorful than that of intact cockerels and is; therefore, produced for specialized market [1–3]. Capon meat is more tender that of cockerels because caponization results in greater increases in subcutaneous, intercellular, and abdominal fat compared with fat accumulation in intact cockerels [4, 5], which improves meat flavor.

Caponization has been shown to cause a significant reduction in testosterone levels in roosters. In male mammals, androgen levels play an important role in adipogenesis [6–10]. Until now, the molecular mechanisms of how androgen levels affect lipogenesis in the avian species were not known. Using the Beijing-You (BJY) chicken, a typical local breed in China, an investigation on global gene expression profiles related to lipid metabolism in abdominal fat following caponization was performed using gene expression profiling. Our data clarify the regulatory mechanisms that influence fat accumulation in caponized chickens and provides interesting insights into these processes.

## Methods

### Animal and experimental design

This study was conducted in accordance with the Guidelines for Experimental Animals established by the Ministry of Science and Technology (Beijing, China). All experimental protocols were approved by the Science Research Department (in charge of animal welfare issues) of the Institute of Animal Sciences, Chinese Academy of Agricultural Sciences (Beijing, China). The BJY hatchlings came from conservation stock (Institute of Animal Science, Chinese Academy of Agricultural Sciences, Beijing, China), and all chickens had the same genetic background. The birds were raised in an environmentally controlled room in three-story step cages. Basal diets were formulated based on the National Resource Council (1994) requirements and the Feeding Standards of Chickens established by the Ministry of Agriculture, Beijing, China (2004). Feed and water were provided ad libitum.

Sixty 3-week-old male chicks with similar body weights were randomly divided into two groups (30 individuals per group). Chicks in one group were caponized while the others underwent a sham operation as the control group. The caponization procedure was performed in accordance with previously described methods [4, 11]. All the capons and control chicks were housed together under standard poultry housing conditions.

Ten birds from each of the two groups were euthanized under carbon dioxide anesthesia by severing the carotid artery at 10, 14, and 19 w after caponization or sham operations. Experienced technicians performed the euthanasias. After a 12-h overnight fast with free access to water, and before euthanasias, blood samples were taken from the brachial veins of chickens. Serum was separated and frozen at − 80 °C until sex hormone concentrations were determined. After slaughter, AF was dissected in the same area for all chickens. The AF samples were weighed, snap-frozen in liquid nitrogen, and stored at − 80 °C until use. The remaining AF tissues were removed and weighed, which included the AF pad and fat around the gizzard. The abdominal fat weight (AFW) was recorded, and the AF percentage (AFP, %) was calculated (mass of AF as a percentage of live weight).

### Determination of serum and AF testosterone levels

Serum testosterone and estradiol concentrations were determined using a radioimmunoassay kit (Huaying, Beijing, China) as previously described method [12]. Testosterone levels in AF were also measured using a chicken-specific testosterone ELISA kit (Fangcheng Biological Technology Co., Ltd., Beijing, China). Samples were homogenized at room temperature and centrifuged (1000×*g*, 20 min) at 4 °C to separate the fat from other debris, and the resultant infranatant fluid was frozen immediately at − 80 °C until use. The assay was performed according to the manufacturers instructions after dilution to optimize accuracy.

### RNA extraction

Total RNA was isolated from AF tissues (each sample was about 100-150 mg) and fat cells using a commercially available kit according to the manufacturer’s instructions (Tiangen, Beijing, China). The concentration and purity of RNA were determined by A_260_ and A_260:280_ (A_260:280_ ≥ 1.8 and ≤ 2.0) using a NanoDrop ND-1000 spectrophoto-meter (Nanodrop Technologies, Wilmington, DE, USA). RNA integrity (RIN ≥ 7) was assessed by electrophoresis on a 1% agarose gel and the Agilent 2100 Bioanalyzer (Agilent Technologies, Palo Alto, CA, USA). RNA samples were stored at − 80 °C until they were used.

### Gene expression profiling

Based on ultra-high-throughput sequencing (HiSeq 2500; Illumina, San Diego, CA, USA), gene expression profiling was undertaken by Berry Genomics (Beijing, China) using total RNA from AF samples in three each of capon and control chickens (taken 14 w after caponizations or sham operations). RNA with good integrity was used to construct the library, and the six cDNA libraries were prepared according to Illumina’s instructions and then, sequenced. Raw data were converted to FASTQ files using bcl2fastq. “Clean” data were obtained by filtering and removing low-quality sequences and mapped to the reference chicken genome and genes (Gallus_gallus, Galgal4; available at https://www.ncbi.nlm.nih.gov/assembly/GCF_000002315.3) using TopHat 1.3.2. Gene expression levels were calculated using the RPKM (Reads Per Kb per Million mapped reads) method as described by Mortazavi et al. [13]. Differentially expressed genes (DEGs) between the treatment and control groups were analyzed using the edgeR R package. To screen for DEGs, we used the following criteria: fold change ≥1.5 or ≤ 0.67, with *P* < 0.05.

Gene Ontology (GO) enrichment analysis was performed for features corresponding to DEGs using the DAVID software toolkit. The significance level for GO term enrichment was set with a False Discovery Rate (FDR) adjusted and a *p*-value < 0.05, by the Benjamini and Yekutieli method [14]. The Kyoto Encyclopedia of Genes and Genomes (KEGG) pathway enrichment analysis was performed using the KOBAS 2.0 (kobas.cbi.pku.edu.cn/help.do) [15, 16]. Pathways significantly associated with DEGs were identified using a hypergeometric test from the R packages (*P* < 0.05, FDR-adjusted). Pathways with fewer than three known chicken genes were discarded.

### Primary preadipocyte acquisition and testosterone treatment

After the primary preadipocytes were obtained from the AF of chickens (2–4 weeks old; Institute of Animal Science, CAAS) according to a previously reported protocol [17], cells were cultured in DMEM/F12 containing 10% FBS. At 100% confluence, cells in three replicate wells of six-well culture dishes were treated with either 0 or 30 ng/mL testosterone. Concurrently, cell differentiation was induced with 10 mg/mL insulin, 0.5 mmol isobutylmethylxanthine, and 1 mmol dexamethasone for 2 d, followed by a 2 d treatment with medium containing 10% FBS and 10 mg/mL insulin, and then with medium containing only 10% FBS. Four days after treatment, cells at a density of 10^6^ per milliliter, as measured by a globulimeter, were used for cell differentiation assays, and for determination of *RXRG*, *FABP7,* and *FABP3* expression in three replicate wells, respectively. RNA extraction from cells was similar to that performed for the AF. Total RNA was also prepared and stored for the quantitative polymerase chain reaction (qPCR) assays.

### The cell differentiation assay

For Oil Red-O staining, cells were fixed in 4% paraformaldehyde for 30 min and washed twice with PBS. They were then immersed in a 0.3% Oil Red-O solution for 2 h. After removing the background Oil Red-O staining with three PBS washes, the stained triglyceride droplets were visualized and photographed. The Oil Red-O staining was then eluted with 100% isopropanol in each well. The eluant absorbance was quantified using a microplate reader at 510 nm. For each group, three replicate wells were used for experimental accuracy. Preadipocyte cultures and differentiation assays were also performed in triplicate.

### Real-time quantitative PCR (qRT-PCR)

Using the same RNA samples as used for gene expression profiling*,* qRT-PCR was performed to confirm the results of gene expression profiling, and 11 representative genes related to lipid metabolism were selected. In addition, in cells treated with 0 or 30 ng/mL testosterone, the *RXRG*, *FABP7,* and *FABP3* expression levels were also detected. The RNA samples were reverse-transcribed using M-MLV reverse transcriptase (Invitrogen, Carlsbad, CA). The specific primers (Table 1) were designed to yield a single product that was verified by a single melting curve peak and a single band on an agarose gel of the appropriate size. The PCR mixture contained 10 μL of 2× iQ™ SYBR Green Supermix, 0.5 μL (10 mmol) of each primer, and 1 μL of cDNA, along with ddH2O making a total volume of 20 μL. Samples were amplified using the real-time PCR Detection System ABI 7500 (Applied Biosystems, Shanghai, China). After initial denaturation for 30 s at 95 °C, amplification was performed for 40 cycles (95 °C for 5 s and 60 °C for 32 s). PCR efficiency for these genes and β-actin was consistent. To determine fold-changes in gene expression, the comparative CT method was used [18], with fold-change calculated as 2^−ΔΔCT^. The results are expressed as the mean fold-change in gene expression from triplicate analyses, using control group samples as the calibrators (arbitrarily assigned an expression level of 1 for each gene). Negative controls, without a cDNA template, were included in this experiment. Correlations between relative abundances from qRT-PCR and gene expression profiling data were also calculated.

**Table 1:** Specific qRT-PCR primers used in this study

Gene name	Sequence	Product size (bp)	Accession NO.
*RXRG*	F:5’-AGGCGTGGGCTCCATCTTT-3’	259	NM_205294
	R:5’-CCGTAGTGCTGGCAGGC-3’		
*FADS2*	F:5’-CTGAGGAAGACAGCAGAGGACAT-3’	153	NM_0011604282
	R:5’-GCAGGCAAGGATTAGAGTTGTG-3’		
*FABP7*	F:5’-CGTGATCAGGACTCAGAGCA-3’	158	NM_205308
	R:5’-TCTCTTTGCCATCCCATTTC-3’		
*SCD*	F:5’-GGCTGACAAAGTGGTGATG-3’	137	NM_204890
	R:5’-GGATGGCTGGAATGAAGA-3’		
*FABP3*	F:5’-AGTACATGAAGGCGTTGGGG-3’	171	NM_001030889
	R:5’-CCGTGGTCTCATCGAACTCC-3’		
*APOA1*	F:5’-GTGACCCTCGCTGTGCTCTT-3’	217	NM_205525
	R:5’-CACTCAGCGTGTCCAGGTTGT-3’		
*MSMO1*	F:5’-TTCGTACTTCTTGCTCTGCGT-3’	226	NM_001006438
	R:5’-CCACCTAGGCATCTCTTCCC-3’		
*SQLE*	F:5’-CGCTGACGGTTGTAGCTGAT-3’	200	NM_001194927
	R:5’-AAGGACGCGAGTCTCAGTTG-3’		
*MGLL*	F:5’-GGAGTCCTCAAAACATCCCATAC-3’	189	NM_001277142
	R:5’-CAAACAAGTTAAGTTCTGTCAGCCT-3’		
*PTGDS*	F:5’-GACGGACAAGTGTATGGCAG-3’	118	NM_204259
	R:5’-CCAGGAAGTAAAGGTGGATATGG-3’		
*CYP2D6*	F:5’-GATTGGAAGAAACCGACCGC-3’	170	NM_001195557
	R:5’-TTGTCGTCCCTTTGGGAATG-3’		
*β-actin*	F:5’-GAGAAATTGTGCGTGACATCA-3’	152	NM_205518
	R:5’-CCTGAACCTCTCATTGCCA-3’		

### Statistical analyses

Statistical analyses between control and treatment groups were performed using Student’s unpaired *t*-test in SPSS Version 16.0 (SPSS Inc., Chicago, IL, USA). Values are reported as the mean ± standard error of the mean (SEM). Differences were considered statistically significant at *P* < 0.01 or *P* < 0.05.

## Results

### Caponization accelerates AF deposition

**Fig. 1:**
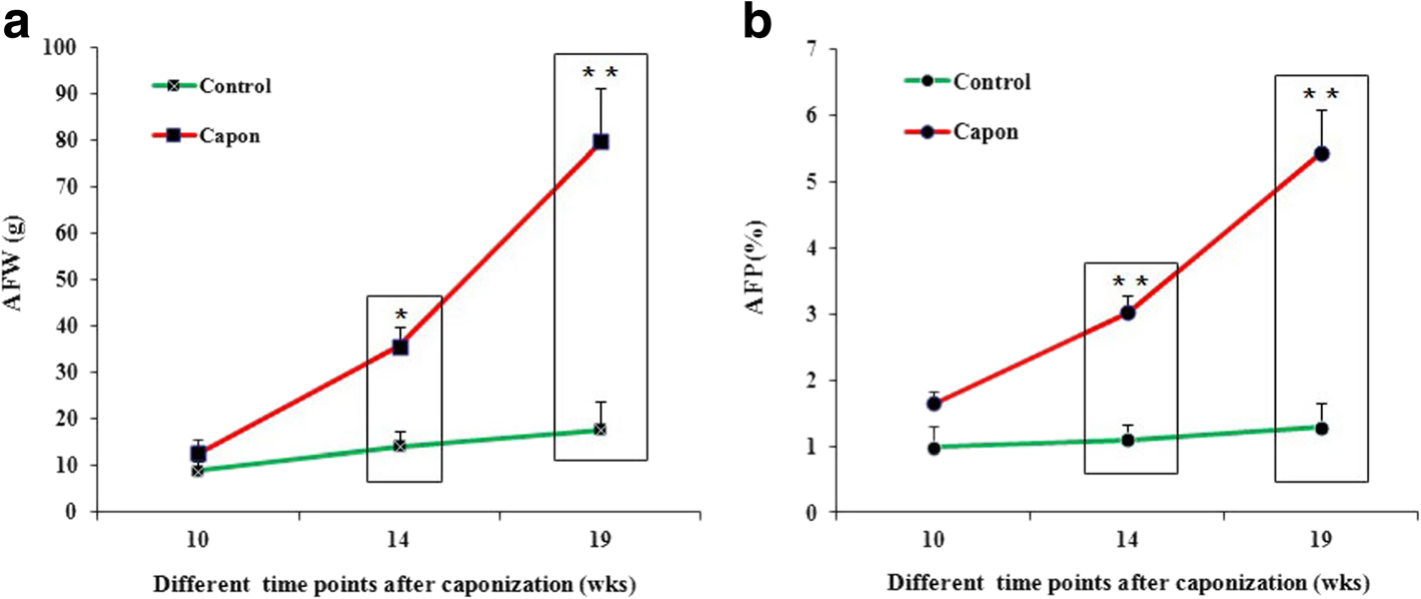
AFW and AFP increase in the abdominal fat of male chickens after caponization. **a** Caponization accelerates AFW in male chickens. The AFWs of Chickens after caponization (capons) or not (sham-operated chickens), were recorded at 10, 14, 19 wks, respectively. The results show that AFW significantly increases in capons compared with control chickens at 14 wk. and 19 wk. after caponization. **b** Caponization improves AFPs in male chickens. AFP (%) is expressed as a percentage of AFW/live weight and significantly increased in capons compared with sham-operated control chickens at 14 wk. and 19 wk. after caponization. Data are presented as means ± SEM, *n* = 10. *P < 0.05; **P < 0.01

With the passage of time after caponization, increases in both AFW and AFP were gradually accelerated. Compared with the sham-operated control animals, AFW and AFP increases were greater in capons (*P* < 0.05 or *P* < 0.01) at 14 and 19 wk. after caponization (Fig. 1a and b). These results suggest that the caponization enhanced AF deposition. And, 14 wk. after caponization, serum and AF samples were used for subsequent experimentation.

### Differential AF gene expression 14 weeks after caponization

To understand the changes in AF deposition in capons compared with controls, AF samples were obtained 14 w after caponization to screen for potential candidate genes that affect AF accumulation. Using gene expression profiling, a total of 872 DEGs (log2 > 0.58 or < − 0.58, with *P* < 0.05) between capons and control group chickens were screened: 463 were up-regulated and 409 were down-regulated in capons compared with controls (Additional File 1).

**Fig. 2:**
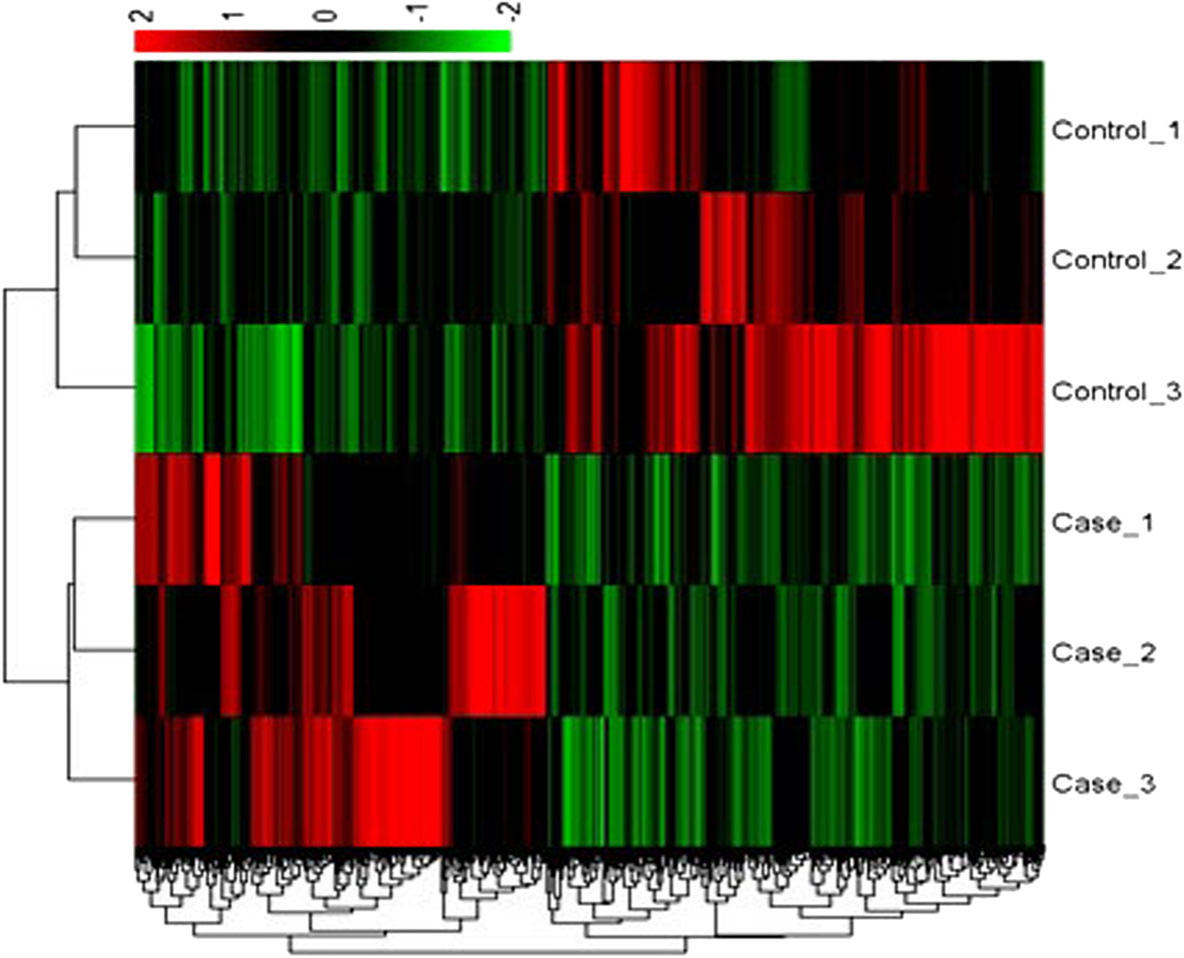
Cluster analysis of AF tissue of capons vs. sham-operated control chickens . **a** Cluster analysis heat-map data using the gene expression profiling. Using Cluster 3.0 software and based on the 872 known DEGs in the AF tissue of capons vs. sham-operated control chickens 14 wk. after caponization, heat-maps of all six samples demonstrated that the three samples in each the capon and the control groups were clustered together, respectively

**Fig. 3:**
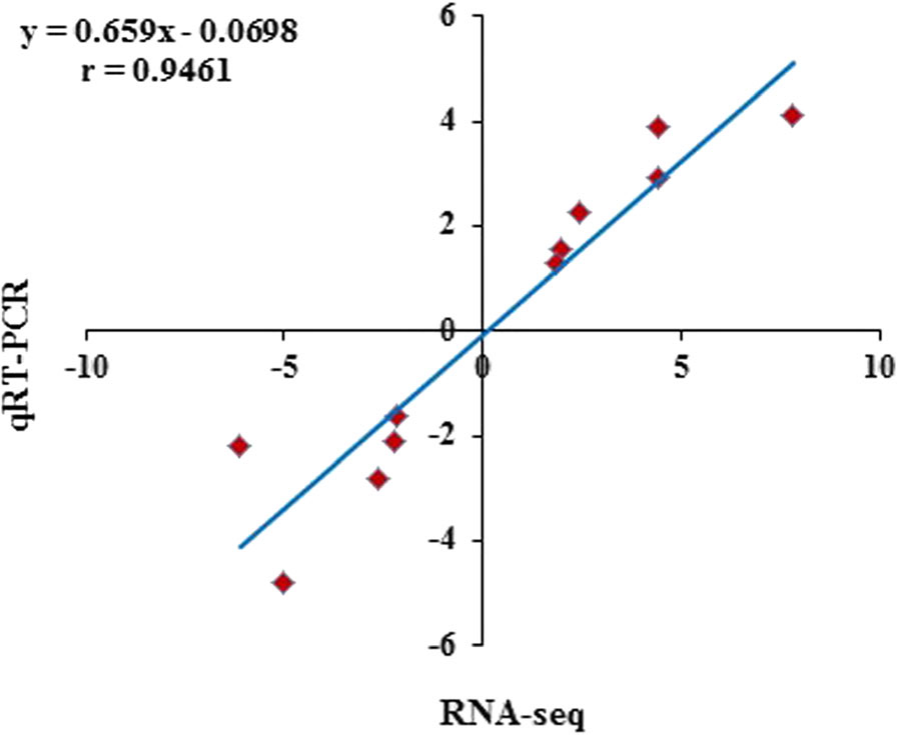
Data verification from Gene Expression Profiling analysis by Q-PCR. **b** The correlation analysis examined the fold-changes of 11 DEGs measured by gene expression profiling and Q-PCR to validate the gene expression profiling results. The data were highly correlated (*r* = 0.9461, *P* < 0.01), indicating that the results from the gene expression profiling were accurate (*n* = 11)

Based on known DEGs, a cluster analysis of all six samples was performed to validate data from the gene expression profiling using Cluster 3.0 software, and it revealed that three samples each from the same group were clustered together (Fig. 2). To validate the data obtained from gene expression profiling, 11 representative DEGs were selected to examine the relative expression using qRT-PCR. As shown in Fig. 3, the fold changes of expression of these genes by gene expression profiling and qRT-PCR were highly correlated (*r* = 0.9461, *P* < 0.01).

GO analysis was performed based on known DEGs and identified particular GO terms in the main biological processes category, included the following processes: lipid metabolism, regulation of cell differentiation, cell cycle, protein metabolism, hormone metabolism, transmembrane transport, oxidation-reduction, muscle system, regulation of the immune system, blood circulation, regulation of apoptosis, and ATP biosynthesis (Additional File 2). Moreover, GO enrichment analysis indicated that the largest proportion of DEGs was involved in metabolic pathways, and 90 genes were associated with lipid metabolism, including 42 up-regulated and 48 down-regulated genes (Additional File 3).

### AF gene expression is enriched for pathways involved in lipid metabolism after caponization

Based on the known DEGs, KEGG analysis was performed, and the 9 significantly enriched (*P* ≤ 0.05) pathways were screened (Additional File 4, Fig. 4a), including well-known pathways related to lipid metabolism (PPAR signaling, arachidonic acid metabolism, and fatty acid metabolism) and cell junctions (ECM–receptor interaction, cell adhesion molecules (CAMs), cytokine-cytokine receptor interaction, and focal adhesion).

**Fig. 4:**
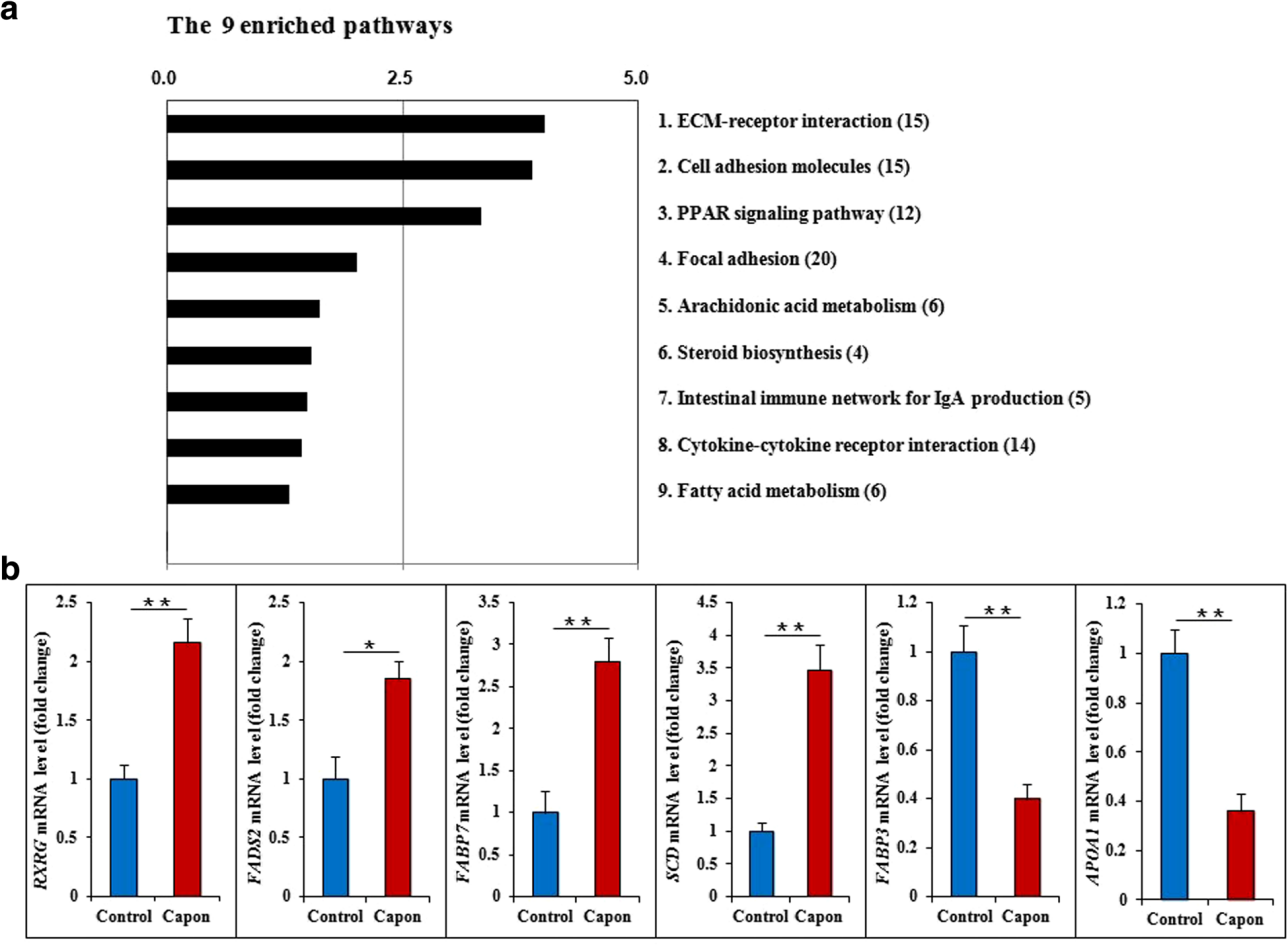
Enrichment of candidate pathways related to lipid metabolism and verification of related genes **a** The nine enriched pathways were screened based on 872 known DEGs. KEGG pathway enrichment analysis was performed using KOBAS 2.0. The enriched pathways were identified using a hypergeometric test from the R packages (*P* < 0.1, FDR-adjusted), and pathways with fewer than three known chicken genes were discarded. **b** The changes in expression of six representative genes involved in the PPAR signaling pathway. The mRNA levels of *RXRG*, *FADS2*, *FABP7,* and *SCD*, which promote lipid deposition, were significantly up-regulated in AF tissues of capons compared with sham-operated control chickens 14 wk. after caponization. *FABP3* and *APOA1* mRNA levels, genes involved in inhibition of lipid deposition, were significantly down-regulated. Data are means ± SEM, *n* = 6. *P < 0.05; **P < 0.01

As for the PPAR pathway, 11 genes related to lipid metabolism, including 6 representative genes (apolipoprotein A1, APOA1; fatty acid-binding 3, FABP3; fatty acid-binding 7, FABP7; retinoid X receptor-gamma, RXRG; stearoyl-CoA desaturase, SCD; fatty acid desaturase 2, FADS2), were specifically associated with the PPAR signaling pathway (Additional File 5: Figure S1), and the mRNA levels of these genes changed significantly (*P* < 0.05 or *P* < 0.01) in the capons compared with controls (Fig. 4b), suggesting that caponization induces gene expression changes that regulate AF deposition.

### Decreased testosterone levels influence AF deposition after Caponization

**Fig. 5:**
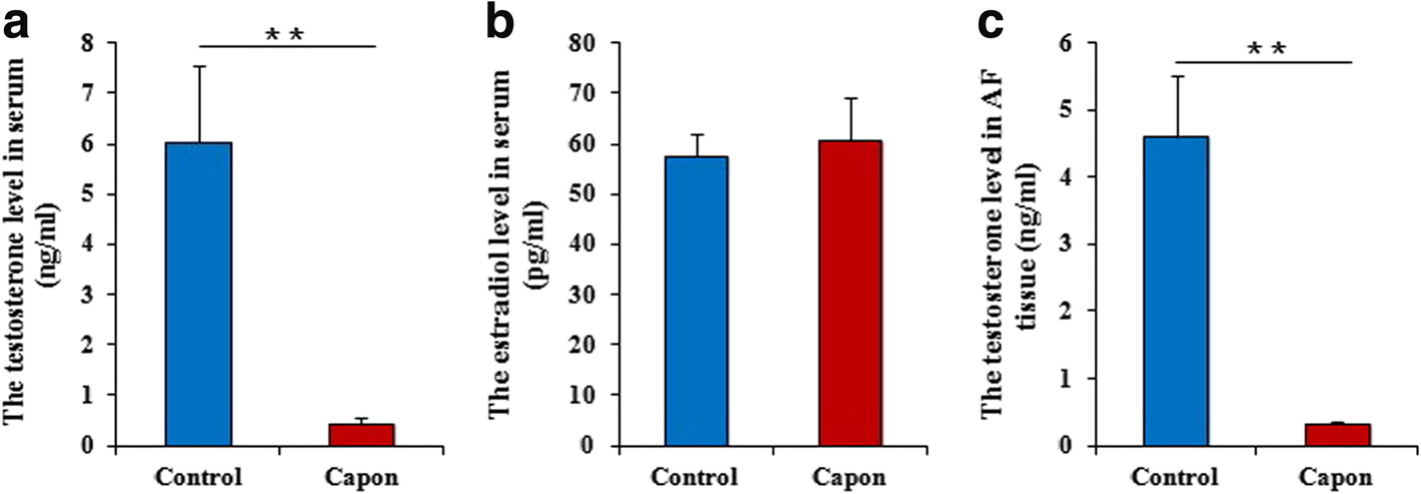
Sex hormone levels in capons vs. control chickens. **a** and **(c)** Serum and AF testosterone levels were significantly reduced in capons compared with sham-operated control chickens. 14 wk. after caponization, testosterone levels were measured with an ELISA kit. **b** Serum estradiol levels were not different between the capons and the control chickens. 14 wk. after caponization, serum estradiol levels were detected with an ELISA kit. Data are means ± SEM, n = 10. *P < 0.05; **P < 0.01

Given that the role of sex hormones in lipid metabolism was previously reported [4], the relationship between testosterone and estradiol and AF deposition was also explored here. First, the serum levels of testosterone and estradiol were determined. As shown in Fig. 5a, serum testosterone levels significantly decreased (*P* < 0.01) 14 wk. after caponization in the capon group compared with those of the control group. In contrast, serum estradiol levels did not differ (*P* > 0.05) between the two groups 14 wk. after caponization or sham operations (Fig. 5b).

Fourteen wks after caponization, chicken AF testosterone levels were determined using an ELISA. As shown in Fig. 5c, the results showed that capon AF had lower testosterone levels (*P* < 0.05) compared with control AF, suggesting that the reduction in serum and AF testosterone levels might correlate with greater AF deposition.

**Fig. 6:**
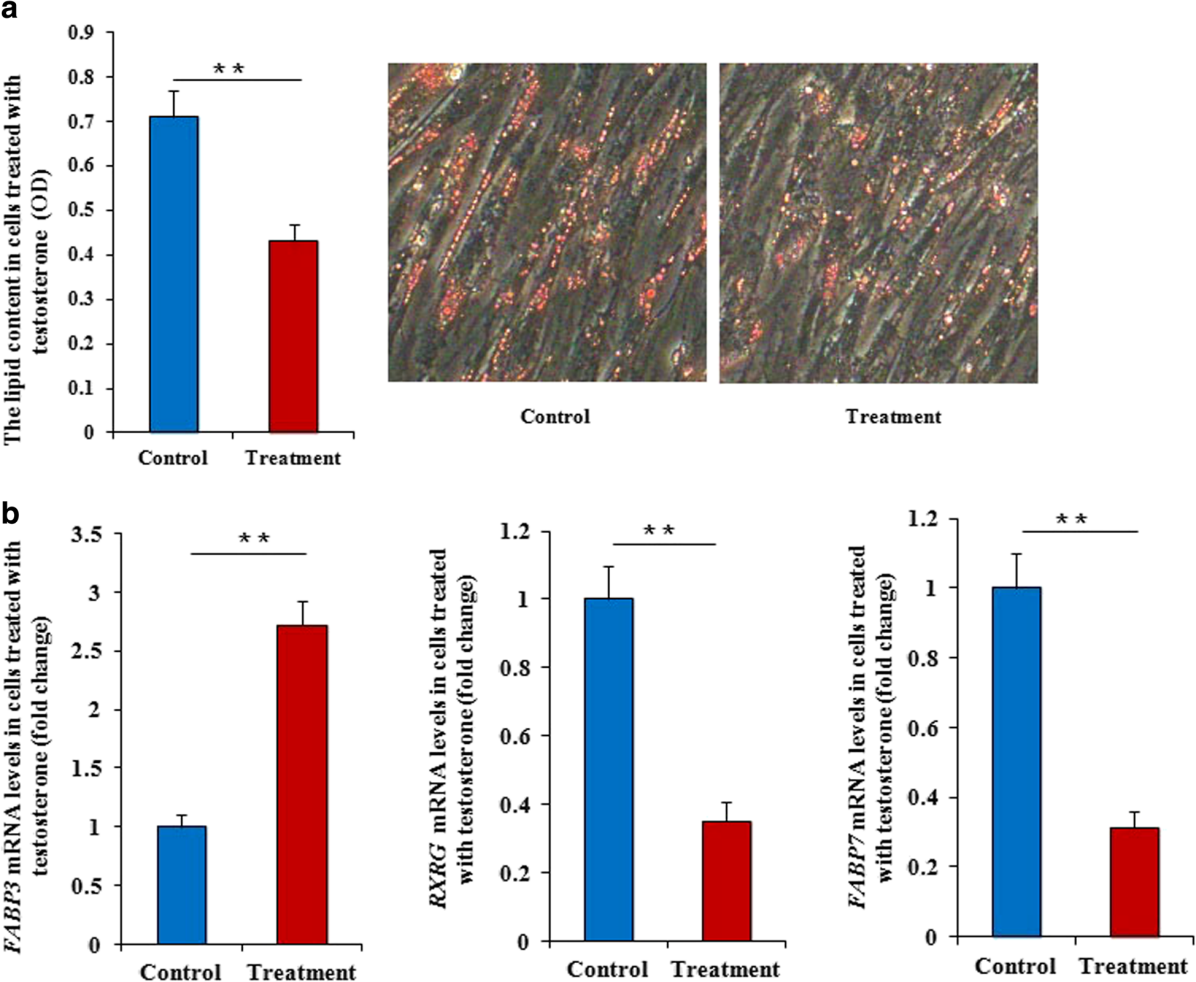
The inhibition of lipid depositon by testosterone in vitro. **a** Testosterone inhibits lipid deposition in primary chicken preadipocytes. Primary preadipocytes were obtained from AF tissues of male chickens, and were cultured with 0 or 30 ng/mL testosterone for 4 d, after which cells were collected, and cell differentiation was determined by oil-red-O staining. **b** *RXRG* and *FABP7* mRNA expression were significantly down-regulated in cells treated with 30 ng/mL testosterone compared with untreated cells. However, *FABP3* mRNA expression was significantly up-regulated. Data are means ± SEM, *n* = 3. *P < 0.05; **P < 0.01

An additional in vitro experiment (with 0 or 30 ng/mL testosterone) was performed to confirm the effect of testosterone on AF deposition. Preadipocyte cell differentiation was detected by Oil Red-O staining after cells were cultured and treated with testosterone or not for 4 d. The fat content in cells treated with testosterone was significantly lower (*P* < 0.01) than in untreated cells (Fig. 6a). Similarly, the expressions of *RXRG*, *FABP3,* and *FABP7* (the important factor involved in the PPAR signaling pathway) were also detected, which showed that *RXRG* and *FABP7* mRNA levels were significantly down-regulated (*P* < 0.01, *P* < 0.01), and *FABP3* mRNA levels were significantly up-regulated (*P* < 0.01) in cells treated with testosterone for 4 d compared with untreated cells (Fig. 6b).

## Discussion

Testosterone, an important androgen, is a major determinant of body composition in male mammals [8, 19]. It has been reported that caponization accelerates AF deposition in male chickens, which is accompanied by decreased testosterone levels [4, 5]. Similarly, abdominal obesity in men is usually associated with a low serum testosterone level [8, 20]. Therefore, it was speculated that caponization would induce fat accumulation by reducing testosterone levels in male chickens, although the molecular mechanisms behind this hypothesis have remained incompletely understood. It is known that intramuscular fat (IMF)has high genetic correlations with AFW and moderate correlations with AFP in chickens [21]. As AF changes, it can change IMF [22–24], which plays an important role in meat quality. Considering the importance of caponization to chicken meat quality, this study was thus intended to reveal the molecular mechanisms behind fat accumulation associated with caponization in chickens.

Using capons, the findings of this study showed that serum testosterone levels were significantly reduced with greater AF accumulation in caponized chickens compared with controls, which is consistent with previous observations [4, 25]. Testosterone, as a circulating blood hormone, can reach distant target tissues to exert its biological functions [7, 10]. The findings indicate that reduced serum and AF testosterone levels might be related to AF deposition after caponization in male chickens. Further, the addition of testosterone to preadipocytes significantly decreased cellular fat deposition. Remarkably, both in vitro and in vivo studies reached the same conclusions, which show that caponization accelerates AF deposition because of reduced testosterone levels.

Based on the results of AF deposition and reduced serum testosterone levels after caponization, the AF tissue samples from capons and sham-operated controls were subjected to gene expression profiling to screen for DEGs and the enriched pathways after caponization in capons versus control chickens. The results of cluster analysis and qRT-PCR supported the accuracy of the gene expression profiling.

GO and KEGG analyses based on 872 known DEGs were performed, and 90 DEGs related to lipid metabolism were further screened, including *RXRG*, *FABP3*, and *FABP7* [26–29]. Among the nine enriched pathways, classical pathways related to lipid metabolism (PPAR and fatty acid metabolism) were screened, and six representative DEGs related to lipid metabolism (*APOA1*, *SCD*, *FABP3*, *FABP7*, *RXRG,* and *FADS2*) were enriched and identified in the PPAR pathway. Considering the importance of the PPAR pathway and the genes related to lipid metabolism [30–35], it was shown that caponization-accelerated AF deposition might be associated with the changed expression of related genes through the PPAR pathway in the AF of chicken. Guo et al. found that caponization increased abdominal fat percentages in Guang-xi Yellow roosters and that *FAS, LPL,* and *PPARγ* mRNA expression levels of *FAS, LPL* and *PPARγ* genes, which are associated with the PPAR signaling pathway, were significantly up-regulated [36]. For the DEGs, the reasons for these discrepancies could be attributed to breed differences and age at the time of slaughter. In addition, pathways related to cell junctions (ECM–receptor interactions, CAMs, cytokine-cytokine receptor interactions, and focal adhesions) were enriched in this study, which showed that these pathways promoted activation of the PPAR pathway to induce AF deposition in chickens, consistent to what was shown in the study by Cui et al. [37]. In the present study, these findings support the assertion that pathways related to cell junctions are also involved in testosterone the regulation of AF deposition.

**Fig. 7:**
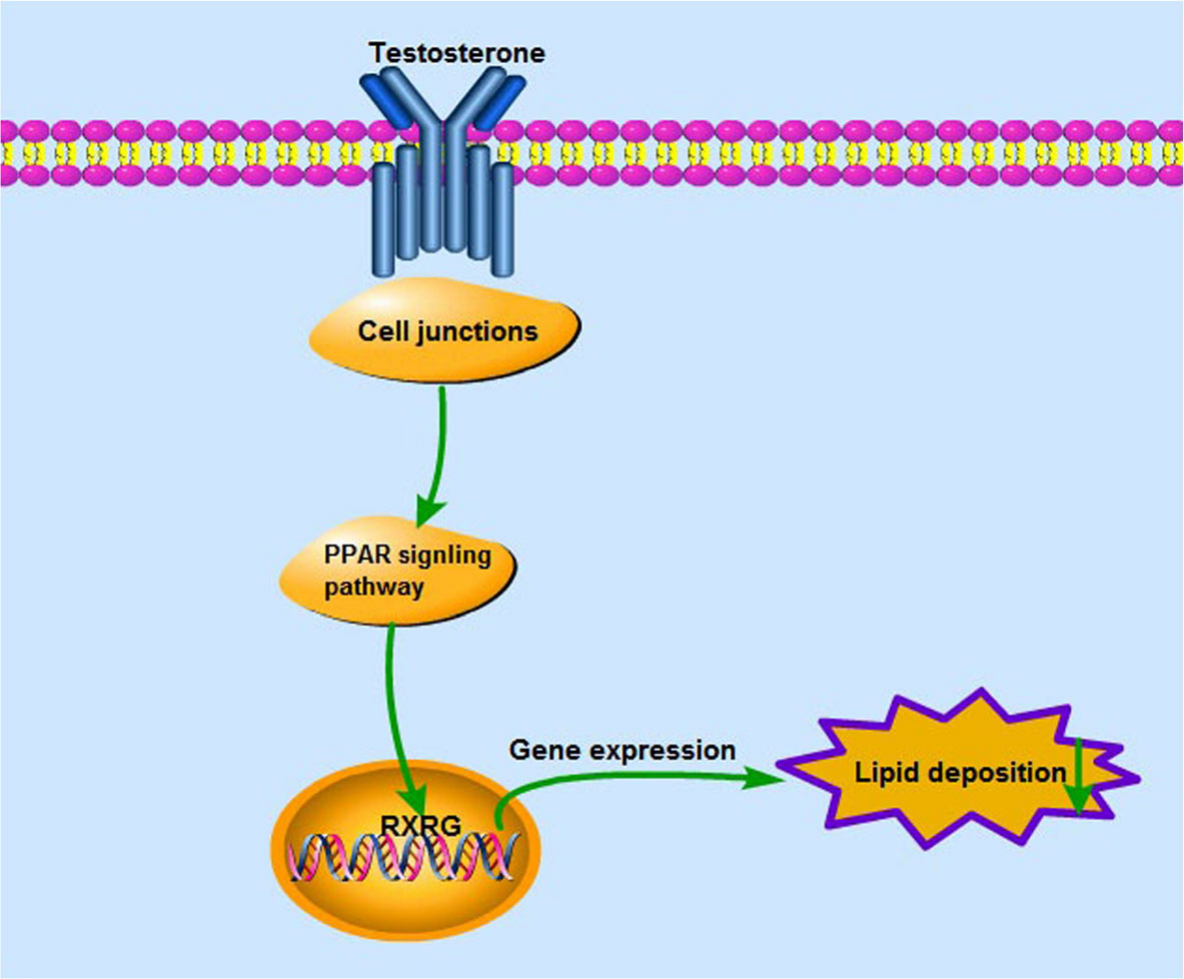
The regulatory network regarding the influence of reduced testosterone levels on lipid deposition in caponized chickens, which is based on significantly enriched KEGG pathways This network is involved in the cellular functions of lipid metabolism (PPAR signaling pathway) and cell junctions.

An additional in vitro experiment (with 0 or 30 ng/mL testosterone) was performed in this study to confirm the effect of testosterone on fat deposition. Cell differentiation was detected by Oil Red-O staining after cells had been treated for 4 d, and the fat content in treated cells was significantly lower (*P* < 0.01) than in untreated ones. Similarly, the expressions of RXRG, FABP3 and FABP7 (the important factor involved in the PPAR signaling pathway) was also detected. The results showed that RXRG and FABP7 mRNA levels were significantly down-regulated (*P* < 0.01, *P* < 0.01), and FABP3 mRNA level were significantly up-regulated (*P* < 0.01) in cells treated with testosterone for 4 d compared with those in untreated cells.Together, these analyses demonstrate that pathways related to cell junctions (focal adhesion, ECM–receptor interaction, cytokine-cytokine receptor interaction, and CAMs), and PPAR might form networks with pathways related to lipid metabolism to influence the AF deposition in male chickens after caponization (Fig. 7).

## Conclusions

In conclusion, our study hypothesized that reduced testosterone levels after caponization accelerates AF deposition by changing the expression of related genes through cell junctions and PPAR pathways in the AF of male chickens. These findings provide a useful foundation for deciphering the molecular mechanisms underlying AF deposition in capons. Additional studies of the associated translational and post-translational effects are required to complement these mRNA expression analyses.

## Additional files

Additional file 1: 872 DEGs between the capon and the control chicken groups. (XLS 311 kb)

Additional file 2: The enriched the GO-terms in the BP category based on 872 DEGs. (XLS 50 kb)

Additional file 3: The 86 DEGs related to lipid metabolism using GO-terms analysis based on a total of 872 DEGs 45 up-regulated and 41 down-regulated. (XLS 75 kb)

Additional file 4: The enriched pathways based on the 872 DEGs. (XLS 61 kb)

Additional file 5: **Figure S1.** The PPAR signaling pathway (JPG 109 kb)

## Abbreviations

AF: Abdominal fat
AFP: Percentage of AFW
AFW: Abdominal fat weight
APOA1: Apolipoprotein A1
BJY: Beijing-You
CAMs: Cell adhesion molecules
CT: Cycle threshold
CYP2D6: Cytochrome P450 2D6
DEG: Differentially expressed gene
FABP: Fatty acid binding protein
FADS2: Fatty acid desaturase 2
FBS: Fetal bovine serum
GO: Gene Ontology
IMF: Intramuscular fat
KEGG: Kyoto Encyclopedia of Genes and Genomes
MGLL: Monoacylglycerol lipase
MSMO1: Methylsterol monooxygenase 1
PPAR: Peroxisome proliferator-activated receptor
PTGDS: Prostaglandin D synthase
qRT-PCR: Real-time quantitative polymerase chain reaction
RXRG: Retinoid X receptor-gamma
SCD: Stearoyl-CoA desaturase
SQLE: Squalene monooxygenase
