# Supplementary figures and images for: Decreased testosterone levels after caponization leads to abdominal fat deposition in chickens
Source: BMC Genomics. 2018 May 9;19:344. doi: 10.1186/s12864-018-4737-3 (PMC5944178; doi:10.1186/s12864-018-4737-3)

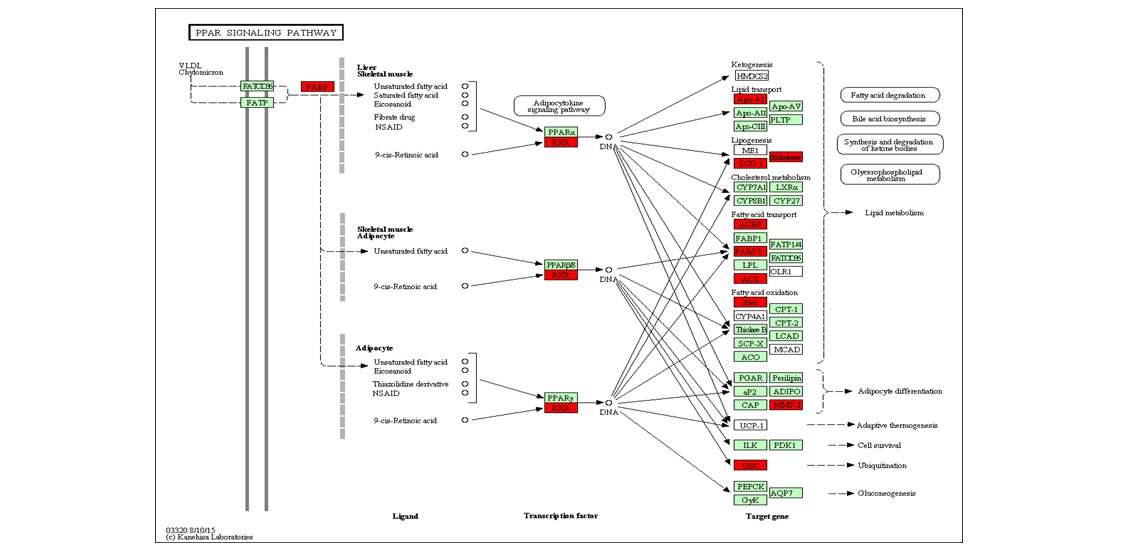

Supplement: Supplementary file 5 — Figure S1. The PPAR signaling pathway (JPG 109 kb) [file 12864_2018_4737_MOESM5_ESM.jpg]
